# Battery Powered Portable Thermal Cycler for Continuous-Flow Polymerase Chain Reaction Diagnosis by Single Thermostatic Thermoelectric Cooler and Open-Loop Controller

**DOI:** 10.3390/s19071609

**Published:** 2019-04-03

**Authors:** Di Wu, Wenming Wu

**Affiliations:** Changchun Institute of Optics, Fine Mechanics and Physics (CIOMP), Chinese Academy of Sciences, Changchun 130000, China; wudi16@mails.ucas.ac.cn

**Keywords:** thermoelectric cooler (TEC), open-loop control, portable continuous-flow PCR, microfluidics

## Abstract

Temperature control is the most important and fundamental part of a polymerase chain reaction (PCR). To date, there have been several methods to realize the periodic heating and cooling of the thermal-cycler system for continuous-flow PCR reactions, and three of them were widely used: the thermo-cycled thermoelectric cooler (TEC), the heating block, and the thermostatic heater. In the present study, a new approach called open-loop controlled single thermostatic TEC was introduced to control the thermal cycle during the amplification process. Differing from the former three methods, the size of this microdevice is much smaller, especially when compared to the microdevice used in the heating block method. Furthermore, the rising and cooling speed of this method is much rapider than that in a traditional TEC cycler, and is nearly 20–30% faster than a single thermostatic heater. Thus, a portable PCR system was made without any external heat source, and only a Teflon tube-wrapped TEC chip was used to achieve the continuous-flow PCR reactions. This provides an efficient way to reduce the size of the system and simplify it. In addition, through further experiments, the microdevice is not only found to be capable of amplification of a PCR product from Human papillomavirus type 49 (Genbank ref: X74480.1) and Rubella virus (RUBV), but also enables clinical diagnostics, such as a test for hepatitis B virus.

## 1. Introduction

Polymerase chain reaction (PCR) technology is a method for amplifying a target DNA sequence in vitro. Developed in 1983 by Kary Mullis, PCR technology has been known as one of the greatest and most widely used inventions in medical and biological research [1,2]. It has been used to amplify a single copy or a few copies of a piece of DNA across several orders of magnitude, generating thousands to millions of copies of a particular DNA sequence [3]. At present, PCR technology displays significant functions in various fields, including environment, biology, physics, and medicine [4,5,6,7], etc.

The process for DNA amplification relies on thermal cycling, which consists of three different stages: the denaturation stage, the annealing stage, and the extension stage [8,9].

There are several available methods for conducting the thermal cycling process of continuous-flow PCR reactions, and three of them were widely used: the thermo-cycled thermoelectric cooler (TEC), the heating block, and the thermostatic heater. Recently, a new approach called open-loop controlled single thermostatic TEC was introduced, to control the thermal cycle during the amplification process of continuous-flow PCR reactions.

**Figure 1:**
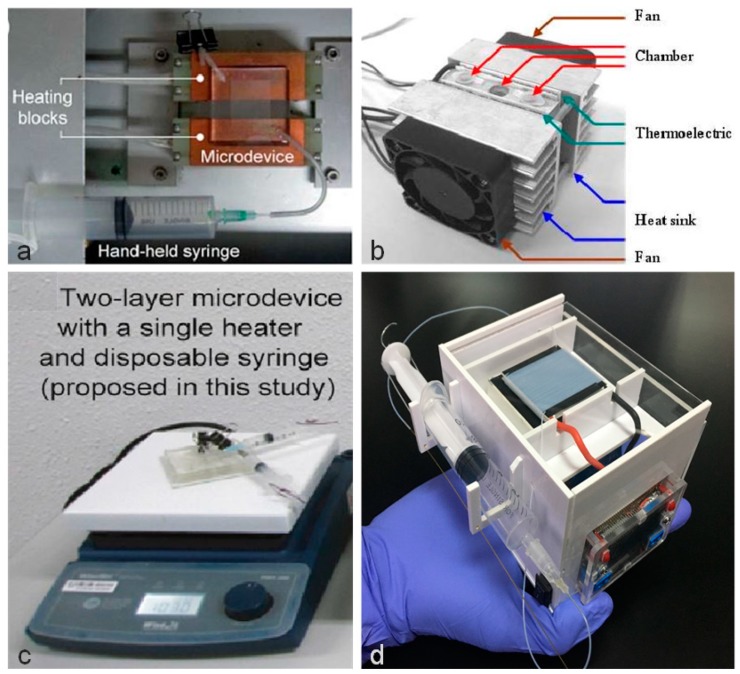
Comparison of the four heating methods. (**a**) By using heating blocks to achieve the required temperature; (**b**) by circuit control to make the thermoelectric cooler (TEC) achieve the required temperature; (**c**) by using a single thermostatic heater to achieve thermal cycling; (**d**) by using a single TEC to achieve thermal cycling of a polymerase chain reaction (PCR) reaction.

Figure 1 shows a comparison of the reaction microdevice and heating block configurations. As shown in Figure 1a, the first method is to use heating blocks to achieve the required temperature [10,11,12]. By placing the microfluidic chip on the heating blocks, which have different temperatures, the microdevice can implement the PCR reaction. Since this system consists of several closed-loop integrated circuits (IC) to control each heating block independently, the circuit control becomes complicated. In addition, the use of several heating blocks makes it more difficult to miniaturize the equipment. The second method is to use the TEC-H bridge controller [13,14]. As shown in Figure 1b, TEC is controlled by a circuit to achieve the denaturation stage and the annealing stage temperatures suitable for a PCR reaction. However, because TEC needs circuit control and a cooling fan, the whole device cannot be miniaturized. In Figure 1c, the third method is to use a single thermostatic heater to achieve thermal cycling [15,16,17,18,19,20]. In order to achieve the suitable temperature, this system requires a closed loop IC to achieve high and low temperature cycle control, which means the temperature change between the denaturation and annealing temperature is slower and circuit control is more complex than the former two methods.

In contrast with the aforementioned three approaches, as shown in Figure 1d, the fourth method was to use a single TEC to achieve thermal cycling of a PCR reaction. What we found in the experiment was that under appropriate circuit control, the TEC can meet the denaturation and annealing temperature requirements at the same time. When the high temperature region reaches 95 °C, the low temperature region reaches 63 °C. What is more, this system only requires an open loop integrated circuit (IC) to maintain the proper voltage, with no need for complex circuit control. Due to its characteristics, the TEC combines several advantages of small size, quick operation, flexible shape, accurate temperature adjustment, no noise, long life, and environmental safety. Because there is no need for complex circuits and heat sinks, the total size of the whole system is reduced.

As far as we know, this miniaturized PCR machinery is believed to be the smallest thermal cycler for continuous-flow PCR reactions, with a weight of 727 g (including a self-contained battery-powered system), and with an option of a DC power supply of only 3.6 V and power consumption of only 6W. This continuous flow PCR system benefits from the novel electronic control, as first introduced here.

Moreover, in the present study, a novel pumping principle and method were used for the passive and velocity-stable transport of liquid. Differing from methods that rely on the permeability coefficient of the end-blocked gas-permeable silicone or PDMS wall [21,22,23,24], an end-opened gas-impermeable quartz tube was adopted to automate the flow. The end-opened microsystem was utilized to replace the blunt-ended microsystems, similar to systems in previous works, for more accurate flow control. The flow rate is fixed under the same pressure, as long as the inner diameter and length of the quartz tube is fixed. The velocity was systemically studied by adjusting the length and inner diameter of the quartz tube, as well as the inner pressure of the fluidic conduit.

## 2. Principle

The TEC uses the Peltier effect to create a heat flux between the junctions of two different types of materials with the consumption of electrical energy, depending on the direction of the current. The device has two sides. When the DC current flows through the device, it induces heat to be transferred from one side to the other, causing one side to become cooler and the other side to become hotter.

**Figure 2:**
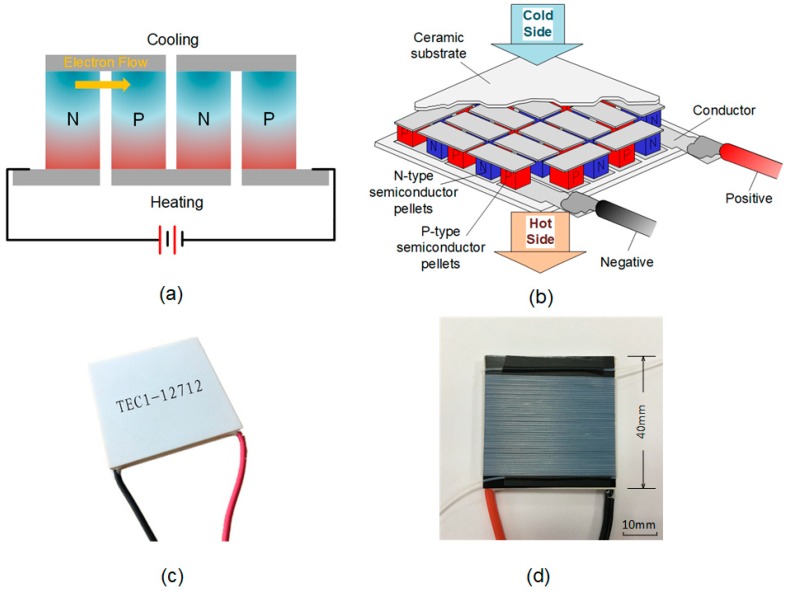
The principle of the thermoelectric cooler (**a**,**b**). An image of the TEC1-12712 type thermoelectric cooler is shown (**c**). The Teflon-wrapped thermoelectric device (**d**).

As shown in Figure 2a,b, the thermoelectric (TE) device comprised of micromodules connected in series. Each micromodule comprised of an n-type and p-type TE semi-conductor material due to different electron densities. The Peltier phenomenon occurs at the junction of these two dissimilar conductors. When a flow of DC current passes through the junction of the semi-conductors, it causes a temperature difference, and the side with the cooling plate absorbs the heat, which is then moved to the other end of the device where the heat sink is located [25].

When the current flows through the interface of two different conductors, heat may be generated (or removed) at the junction. This phenomenon is called the Peltier Effect. Peltier heat is generated at the junction per unit time:

(1) $$\dot{Q}=\left(\Pi_{P}-\Pi_{N}\right)I$$

where $\Pi_{P}(\Pi_{N})$ is the Peltier Coefficient of the p-type and n-type TE semi-conductor material, and I is the electric current that flows through the conductor.

A single-stage TEC will typically produce a maximum temperature difference of 70 °C between the hot and cold sides. Hence, the more heat is moved, the less efficient it becomes. This is due to the need of the TEC to dissipate both the heat being moved as well as the heat it generates itself from its own power consumption. The amount of heat that can be absorbed is proportional to the current and time:

(2) W=ΠIt

where Π is the Peltier Coefficient, I is the current, and t is the corresponding time.

As for the micropump of the system, a single syringe was used as a self-activated micropump. At the end of the system, a quartz tube was attached to the tail Teflon tube. This novel micropump system was studied in our former work [26], which was based on the air permeability from the fluidic conduit to the atmosphere. The permeation actually relies on the air that passes through the hollow channel of the gas-impermeable quartz tube, and not the air passing through the wall of the gas-permeable silicone or PDMS wall, which causes a totally different mechanism and mathematic modeling. Since the pressure of the compressed air captured inside the fluidic conduit is much higher than the atmospheric pressure, air molecules inside the microchip tend to penetrate to the ambient atmosphere through the hollow channel of the quartz tube, which causes a decrease in the mole-number of air molecules in the anterior end of the sample plug. This can be calculated using the following equation.

(3) $$G_{a}=\frac{DA_{ave}}{Z}\left(C_{Aia}-C_{Ao}\right)$$

(4) $$C_{Aia}=\frac{P_{a}}{RT}$$

(5) $$C_{Ao}=\frac{P_{Ao}}{RT}$$

where $G_{a}$ is the diffusion flux, D is equivalent diffusion coefficient, $C_{Aia}$ is inner air molecule concentration in the anterior end of the sample plug, $C_{Ao}$ is the air molecule concentration of the ambient atmosphere, Z is the diffusion length, and $A_{ave}$ is the average diffusion area.

**Figure 3:**
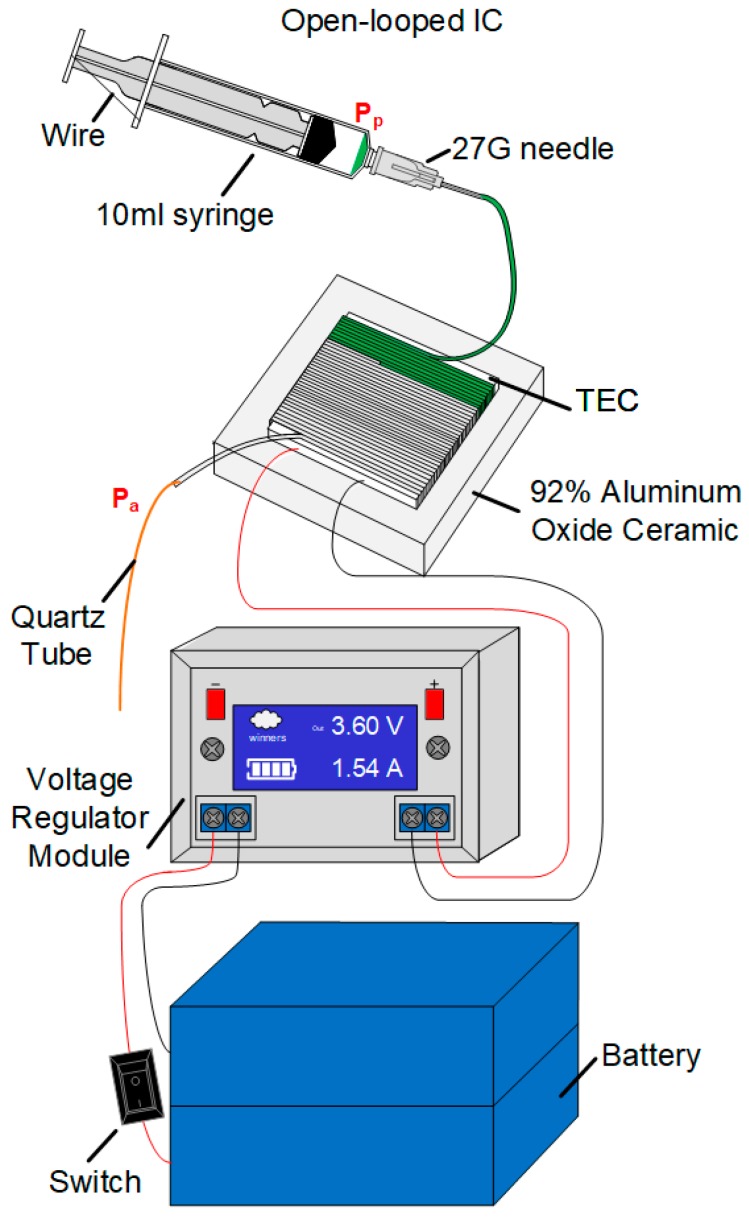
The assembly of the microdevice.

As shown in Figure 3, $P_{p}$ and $P_{a}$ represent the air pressures in the posterior and anterior ends of the reagent, while $P_{g}$ represents the pressure gradient imposed in the reagent. These can be calculated through the following equation:

(6) $$P_{g}=P_{p}-P_{a}$$

## 3. Experimentation

Due to the special characteristic of the thermoelectric device, we decided to use the TEC1-12712 type TEC, as shown in Figure 2c,d. The size of the devices is 40 mm × 40 mm × 3.3 mm, with a rated voltage of DC 12 V. The 12712 model is usually used as a semi-conductor refrigeration unit for water dispensers or refrigerators. The maximum temperature difference is 62 °C. Thus, by changing the input voltage, it is possible to determine the suitable value for the temperature of each side of the TEC, which is capable of the PCR reaction. Moreover, the temperature of the upper high-temperature surface is correlated not only to the input voltage, but also with the kind of upper spacer materials. Thus, in order to determine the appropriate condition, a series of voltage gradient experiments was conducted using three different spacers underneath. According to the thermal conductivity table of various materials, three of these materials were chosen for the test due to the thermal conductivity, showing that these materials could more likely reach the expected conditions. These were as follows: 92% Aluminum Oxide Ceramics, Silicon Carbide Ceramics, and 304 Stainless Steel. The size of these materials was all 50 mm × 50 mm × 10 mm.

**Figure 4:**
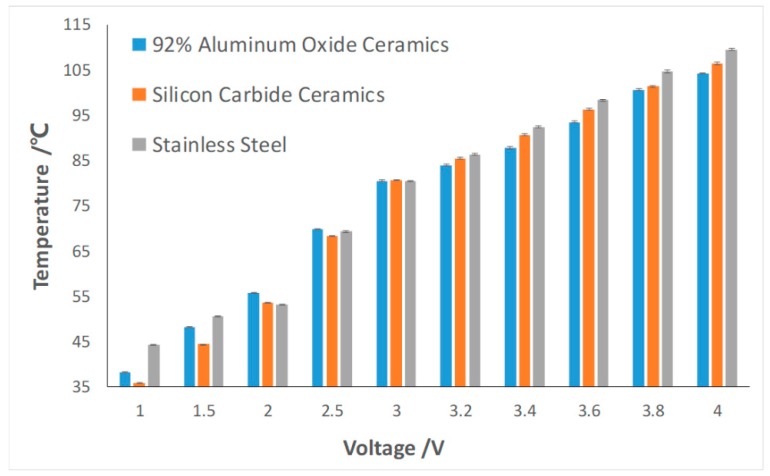
Temperatures of the high-temperature region of the thermoelectric cooler under different voltages and spacers.

A set of voltage gradient experiments for each of the different spacers was conducted. In order to meet the temperature requirement of denaturation stage, we monitored the temperatures of the high-temperature region by adopting different kinds of spacers and setting up different voltages. As shown in Figure 4, the temperature in the high-temperature region increases with the increase in voltage. In order to determine the actual voltage and current values at that moment, an adjustable step-down voltage regulator module, with an input voltage of 5–23 volts and an output voltage of 0–16.5 volts (TELESKY, 3A, DC, 62 mm × 44 mm × 18 mm), was attached to the TEC power system. Since this has the smallest thermal conductivity among the three, the heat transferred to the 304 stainless steel was lesser than that to the other two materials. Therefore, the upper surface temperature was highest among those three spacers under any input voltage. The investigators documented the temperature in the low-temperature region while conducting the gradient experiment, and compiled the data, as shown in Table 1.

As shown in Figure 4 and Table 1, it was relatively a suitable condition for the PCR reaction when a 92% Aluminum Oxide Ceramic was used for the spacer, with a setup voltage at 3.6 V. The high-temperature zone was approximately 95 °C, which was appropriate for the denaturation stage, while the low-temperature zone was nearly 63 °C, which was a suitable temperature for most of the reagents to react. The investigators consider that by balancing the input voltage and the material and thickness of the spacer, the system would be able to achieve most of the conditions that a PCR reaction would require.

**Figure 5:**
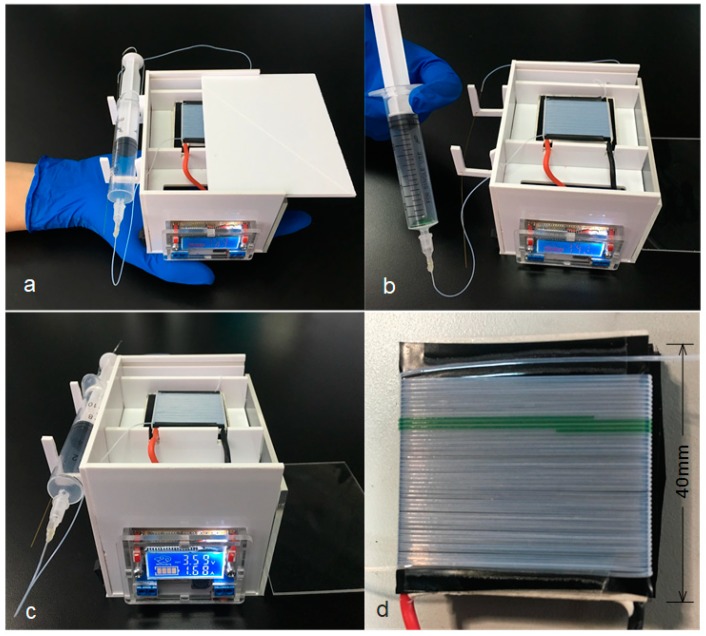
An image of the microdevice (**a**–**c**). The flow situation of the reagent (**d**).

The measurement of the assembled portable PCR device shown in Figure 5a,c was 115 mm × 85 mm × 80 mm. It consists of two 3.7 V polymer lithium batteries connected in series (ZON.CELL, 12,000 mAh, 70 mm × 60 mm × 20 mm), an adjustable step-down voltage regulator module, a ship-shaped switch (TELESKY, 6 A, 250 V, 13.5 mm × 8.5 mm), a 92% Aluminum Oxide Ceramic, a TEC (TEC1-12712), a 10-mL syringe, Teflon tubes (the inner and outer diameters were 0.3 mm and 0.6 mm, respectively), an iron wire, a 27 G needle, and a 15-cm long quartz tube (inner diameter, 25 μm). The whole system was installed in a box made of polymethyl methacrylate (PMMA). The right wall and top of the box were made of transparent PMMA to allow the internal structure to be observed, while the rest of it was made of white PMMA.

In order to operate the system, the switch was turned on, and the voltage regulator module was set-up to 3.6 V. Then, there was a wait of approximately 15 min to preheat the TEC. Next, the reagents were placed on the inner side wall of the syringe, and the syringe was pushed from the scale of 10 mL to the scale of 6 mL. After that, the syringe was positioned upright to allow the reagents to spontaneously flow down under pressure. When all the reagents flowed into the Teflon tube, the syringe was positioned horizontally on a bracket on the left wall of the box. As shown in Figure 5d, the flow time of the reagents was approximately 60 s per cycle to reach a suitable reaction time for a PCR assay, the flow rate was 8.5 μL per minute, and the Teflon tube was wrapped for 40 laps. Tests showed that the microdevice can work stably for three hours with two polymer lithium batteries in series.

In order to prove that the microdevice can be used for continuous flow PCRs, a commercial qPCR cycler (CFX Connect; Bio-Rad, CA, USA) was used as a reference cycler system. By comparing the PCR products from these two devices, the function of this system could be verified. In addition, in order to prove the practicability of the system and determine the PCR amplification efficiency of the microdevice in the present study, two kinds of reagents were used, and these reagents were tested in parallel by both the microdevice and commercial qPCR cycler (Bio-Rad). The reaction condition in the commercial qPCR cycler was set with an annealing stage and denaturation stage of 30 s, 63 °C, and 30 s, 95 °C, respectively. Meanwhile, the flow rate of the reagent in the Teflon tube was approximately 60 s per cycle. Due to the shape of the PDMS block, the 95 °C denaturation temperature and the 63 °C annealing temperature both lasted for 30 s.

The DNA fragment of Human papillomavirus type 49 (Genbank ref: X74480.1), Rubella virus (RUBV), and Hepatitis B Virus (HBV) were amplified using the microreactor. The target amplifications of Human papillomavirus type 49 were 75 bp in size. The target amplifications of RUBV was 88 bp. The primer sequences for amplifying a 75 bp gene fragment of Human papillomavirus type 49 were as follows: 5′-GCCAACCCCTCCAGAAACA-3′ (forward), and 5′-CCCACCTCCACCAGTAAA CG-3′ (reverse). The primer sequences for amplifying an 88 bp gene fragment of RUBV were as follows: 5′ATTGTTATGTATGAGCGGTGA A-3′ (forward), and 5′-TTGTAAAGCCCTATGAGTGAG C-3′ (reverse). The PCR reagent was composed of 1× SYBR Premix Ex TaqII, 0.075 U µL^−1^ TaKaRa EX Taq, 0.6 mg mL^−1^ BSA (AS25483; AMEKO, www.biolianshuo.com, China), 1 µM of forward and reverse primers, and 0.00322 ng µL^−1^ of template. Each test required 40 µL of reagent.The Hepatitis B Virus Nucleic Acid Detection Kit was applied for amplification of HBV gene, which consisted of the primer and probe. After the PCR reaction, agarose powder (V900510; Sigma-Aldrich, www.sigmaaldrich.com, MO; 2%), DL2000 DNA marker (50 × 250 µL, Peking Jialan Biotechnology Co., Ltd., Beijing, China), 0.5 × TBE buffer (PH1755, Phygene, China), and Nucleic Acid GelStain (KeyGEN BioTECH, Nanjing, China) were applied to analyze the amplification result, which was detected at 254 nm with an UV illuminator. Positive control experiments were conducted using a thermal cycler (CFX Connect, Bio Rad, USA) under the same thermal-cycling conditions for all targets, and the high-resolution multiplexed PCRs as well. Denaturation and annealing or extension were performed at 95 °C and 63 °C, respectively, requiring 40 thermal cycles of amplification procedure. Three experiments were carried out for each reagent. The best results are shown below.

## 4. Results and Discussion

**Figure 6:**
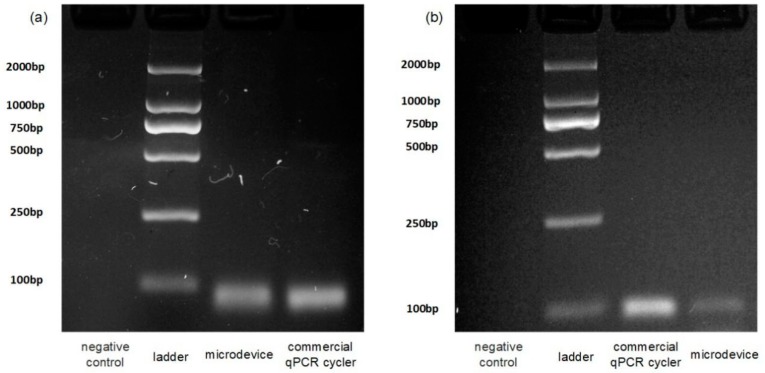
The electrophoretograms of (**a**) the DNA fragment of Human papillomavirus type 49 and (**b**) Rubella virus (RUBV).

**Figure 7:**
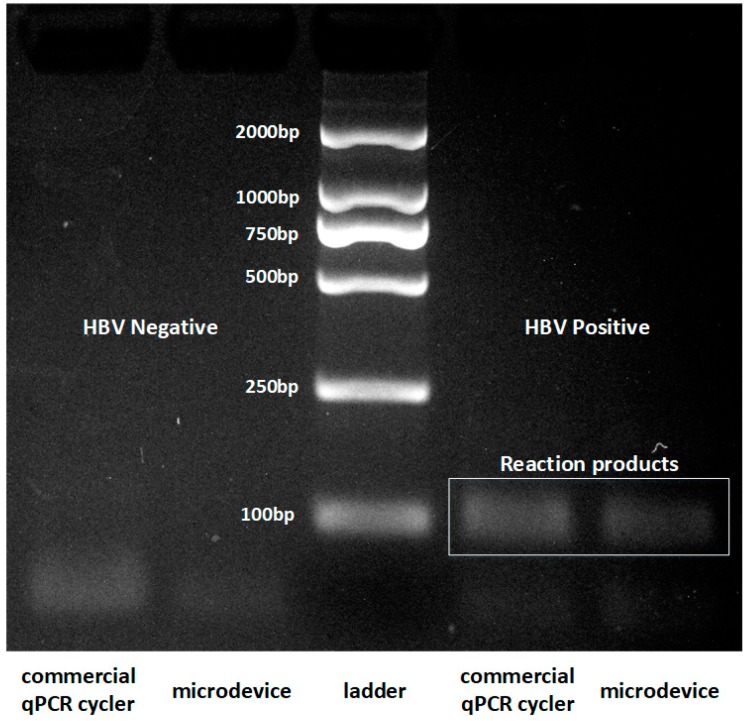
The electrophoretogram of the examination of hepatitis B virus.

As a proof of concept, various DNA targets were performed on the portable PCR platform. Each assay was tested twice and the best results are shown in Figure 6 and Figure 7. Figure 6 shows the amplification results of our microsystem and the Bio Rad thermal cycler. As shown in Figure 6, the intensity of the target amplicons obtained from the polymer lithium battery-powered microreactor is close to that obtained using the commercial thermal cycler. Figure 6a demonstrates the results of the electrophoretic reaction of the DNA fragment of Human papillomavirus type 49 and Figure 6b demonstrates the results of the electrophoretic reaction of Rubella virus (RUBV). As shown in the figure, the two methods of the commercial qPCR cycler (Bio-Rad) and the present system have the same products. This means that the present system is capable of carrying out a PCR reaction. Although the chip gained band is a little weaker, this is inevitable for continuous-flow PCRs because the big inner surface of the Teflon tube can unexpectedly adsorb the biomolecules. After calculation of amplification results of the targets by ImageJ, the average amplification efficiencies are about 78% and 43% of those of a commercial cycler. The results indicate the portable PCR platform can be applied for preliminary on-chip amplifications.

To further test system performance for clinical diagnostic tests, an experiment was conducted to verify whether the present system can be applied for the detection of hepatitis B virus. A Hepatitis B Virus Nucleic Acid Detection Kit provided by Anhui Targene Medical Technology Co., Ltd., was used, and both the HBV positive and HBV negative assays were performed using the commercial qPCR cycler (Bio-Rad) and the present system. The results are shown in Figure 7.

In Figure 7, the ladder is shown in the middle, and the two lanes on the left of the ladder represent the products gained by the commercial qPCR cycler (Bio Rad) and the present system. The two lanes on the right of the ladder were gained by the commercial qPCR cycler (Bio Rad) and the present system. As shown in the figure, the gene targets from HBV positive serum were successfully amplified by both the commercial thermal cycler and the portable PCR platform, respectively, and the HBV negative sample had no products at the position of the product strip through both the commercial qPCR cycler (Bio-Rad) and microdevice. The intensity of the amplified HBV target of the microreactor was approximately 61% of that obtained by using a commercial thermal cycler. Based on this result, it is proven that the polymer lithium battery-powered continuous-flow PCR platform can be applied for diagnosis of HBV. It can provide an easier and more energy-efficient way of performing a PCR reaction.

## 5. Conclusions

In this study, we introduced a novel mechanism, in which a single TEC was used as a thermal cycler and a self-powered end-opened micropump system. This provides a new approach to thermal cycling, in which the Teflon tube was wrapped around the TEC to perform the PCR reaction. The gel images revealed that this portable microdevice is also capable of clinical diagnostic tests. The design of the portable PCR microreactor simplifies the traditional processing method and makes the operation more convenient with general applicability. At the same time, the portable PCR microreactor can provide reliable results for the detection of Human papillomavirus type 49, RUBV, and Hepatitis B Virus (HBV). In future studies, this system will be upgraded to make it more controllable, allowing it to be applied to wider fields.

## Figures and Tables

**Table 1:** Temperatures of the low-temperature region of the thermoelectric cooler under different voltages and spacers.

Voltage (V)	Temperature (°C)		
	92% Aluminum Oxide Ceramics	Silicon Carbide Ceramics	304 Stainless Steel
1	30	30	34
1.5	34	32	36
2	38.5	35	37
2.5	48	46	47
3	56	54	56
3.2	58	57	60
3.4	60	62	63
3.6	63	65	67
3.8	68	69	72
4	70	73	75
